# Continuous and automatic mortality risk prediction using vital signs in the intensive care unit: a hybrid neural network approach

**DOI:** 10.1038/s41598-020-78184-7

**Published:** 2020-12-04

**Authors:** Stephanie Baker, Wei Xiang, Ian Atkinson

**Affiliations:** 1 College of Science and Engineering, James Cook University, Townsville, 4811 Australia; 2 School of Engineering and Mathematical Sciences, La Trobe University, Melbourne, 3086 Australia

**Keywords:** Biomedical engineering, Health care, Prognosis, Risk factors

## Abstract

Mortality risk prediction can greatly improve the utilization of resources in intensive care units (ICUs). Existing schemes in ICUs today require laborious manual input of many complex parameters. In this work, we present a scheme that uses variations in vital signs over a 24-h period to make mortality risk assessments for 3-day, 7-day, and 14-day windows. We develop a hybrid neural network model that combines convolutional (CNN) layers with bidirectional long short-term memory (BiLSTM) to predict mortality from statistics describing the variation of heart rate, blood pressure, respiratory rate, blood oxygen levels, and temperature. Our scheme performs strongly compared to state-of-the-art schemes in the literature for mortality prediction, with our highest-performing model achieving an area under the receiver-operator curve of 0.884. We conclude that the use of a hybrid CNN-BiLSTM network is highly effective in determining mortality risk for the 3, 7, and 14 day windows from vital signs. As vital signs are routinely recorded, in many cases automatically, our scheme could be implemented such that highly accurate mortality risk could be predicted continuously and automatically, reducing the burden on healthcare providers and improving patient outcomes.

## Introduction

Intensive care units (ICUs) treat the most critically ill patients, and as a result are known to have the highest mortality rate of hospital units^1^. In 2001–2012, mortality rates across several ICUs in the United States (US) ranged from 11.3 to 12.6%^2^. As such, typical ICUs have high staff-to-patient ratios, and it has been found that outcomes are improved where there are a higher number of nurses and consultants per bed^3^. However, providing a high standard of critical care comes at a cost, with $108 billion spent on critical care medicine in the US in 2010, accounting for 0.72% of gross domestic product (GDP)^4^. The use of mortality risk assessment tools can aid in resource allocation and treatment decisions, potentially reducing costs while continuing to provide a high standard of care to critically ill patients.

There are several points-based schemes currently used to quantify mortality risk in ICUs today, including multiple iterations of the Acute Physiology and Chronic Health Evaluation (APACHE) score and Simplified Acute Physiology Score (SAPS). While newer versions exist, APACHE-II^5^ and SAPS-II^6^ remain the most commonly used mortality risk assessment tools worldwide^7^. Another commonly used tool is the Sequential Organ Failure Assessment (SOFA) score^8^, which was developed to assess sepsis risk but has since been found to be a relatively good predictor of mortality. Unfortunately, there are several limitations associated with these tools. Firstly, it has been found that their performance decreases fairly rapidly over time, with Kramer^9^ indicating that SAPS II was out of calibration by 2005. Several subsequent studies have also identified calibration problems with APACHE, SOFA, and SAPS^10–12^. Calibration can be lost over time due to changing patient populations and medical treatments, and typically results in overestimation of mortality^9^. Aside from the effect of time, Sakr et al.^10^ and Lew et al.^12^ noted that the schemes performed poorly for European and Singaporean cohorts, respectively. This indicates that insufficient consideration of diverse patient cohorts has also affected performance. Aside from calibration issues, these schemes rely on variables that can be time-consuming and difficult to obtain, such as pathological laboratory test results and patient medical history.

The limitations of existing scoring systems have lead to a rise in researchers exploring machine learning techniques for mortality prediction^13–20^, as well as the related issues of predicting the onset of various intervention methods^21,22^ detecting the risk of sepsis^23–26^ and other clinical deterioration events^27,28^. Machine learning approaches have the advantage of being relatively easy to continuously update and recalibrate, with algorithms able to be configured in a way that enables continuous training based on new data obtained while it is being used in clinical environments. This in turn enables machine learning techniques to better generalise to current local or global populations, even as treatments and outcomes change with time. A recurring theme in these papers is a dependence on features including complex laboratory results, existing health conditions, and other patient history. Of the aforementioned studies that consider mortality risk prediction, the majority depend heavily upon laboratory results^14–19^, which include values obtained from extensive blood, urine, breath, and other clinical analysis, and are often complex and time-consuming to obtain and then enter in patient’s medical records.

A common metric for assessing the discrimination performance of diagnostic tools is the area under the receiver-operator curve (AUROC). In terms of this metric, the highest performing mortality prediction systems were presented by Johnson et al.^17^ and Delehanty et al.^18^ with AUROCs of 0.927 and 0.94, respectively. However, the system presented by Johnson et al.^17^ is dependent on 148 features comprised predominantly of complex laboratory results. This limits the usefulness of the system, as medical staff would need to measure and enter a massive number of variables to receive an accurate prediction.

Meanwhile, in the work presented by Delahanty et al.^18^, only 17 variables were used—however, over 50% of the decision made by their system is based on All Patients Refined Diagnosis Related Groups (APR-DRG) risk of mortality and severity of illness, as well as Glasgow Coma Score (GCS), and the cost-weight index based on Medicare Diagnosis Risk Groups (MS-DRG). In short, this system depends heavily on diagnoses being made manually by doctors based on data available close to the time of ICU admission. This introduces potentially heavy human bias, and would not be functional for hospitals where the APR-DRG and MS-DRG diagnosis coding schemes aren’t used.

Conversely, Deliberato et al.^13^ investigated the use of features extracted from only vital signs, achieving a relatively low AUROC of 0.65, indicating low ability to distinguish between mortality and non-mortality cases. The authors also considered using vital signs in combination with other parameters, achieving a much higher AUROC of 0.84 when vitals data was combined with the GCS, SAPS-II score, patient demographics and information obtained about the patient during their hospital stay prior to ICU admission. This is certainly an improved performance; however it depends upon significant lab results and data from pre-admission. It would be preferable to use only vital signs and basic demographics, but with much higher performance than the 0.65 AUROC achieved by this work.

Another common theme in the literature is that of mortality prediction at admission. While there are advantages of early mortality risk prediction, this method is inflexible and does not consider how a patient might respond to treatments after ICU admission. This was identified in a recent work by K. Yu et al.^20^, where a bidirectional long short-term memory network was trained on multiple windows to identify mortality risk at any given time. This scheme uses the same features such as the SAPS-II score, which includes many laboratory values, GCS, demographic information, admission type, and comorbidities. As such, laboratory measurements would need to be repeated regularly for the system to predict effectively. Additionally, it requires 48 h of data to predict future mortality risk effectively.

In the literature, there are many machine learning (ML) techniques considered for prediction of mortality. However, there have been relatively few that investigate the use of neural networks (NNs) specifically. Early works investigating NNs for mortality prediction focused on simple feed-forward neural networks^29–31^, achieving comparable performance to scoring schemes such as APACHE. Works in recent years have begun to focus on NNs that are more advanced, such as long short-term memory (LSTM) NNs and convolutional NNs (CNNs). Several works^14,20,32^ have identified long short-term memory (LSTM) networks as candidates for mortality prediction. LSTM has also proven successful in predicting septic shock^24^ and other clinical deterioration events^28^. The primary advantage of LSTM is that it has the ability to ‘remember’ information that it has already seen, allowing it to identify relationships between different variables within the sequence.

Meanwhile, convolutional neural networks (CNNs) have also proven powerful in solving many medical problems such as detecting heart anomalies^33–35^, identifying variations in Korotkoff sounds^36^ and gait detection^37^. CNNs are exceptional at identifying the importance of certain features with respect to one another, and thus adding CNN layers prior to LSTM layers can greatly improve the predictive ability compared to pure LSTM. This was attempted by Alvis et al.^15^, with their scheme achieving an AUROC of 0.836 when predicting ICU mortality from a 48-h window of features including vital signs and laboratory values. This strongly suggests that a well-designed CNN-LSTM network would be a good candidate for mortality prediction, combining the benefits of both network types for a resultant network that can identify important variables and any relationships that exist between them.

Aside from the model itself, another critical factor in the success of a neural network is the selection of features. To develop a system that could automatically update mortality risk throughout a patient’s stay, it is essential to choose features that are both meaningful and easy to measure, ideally without any manual measurement. One recent work by Giannini et al.^23^ considered hundreds of features for predicting septic shock, a strong risk factor for mortality. In a retrospective analysis, the authors found that 10 out of 20 of the most important features were derived from vital signs, while another was age. This finding is significant but largely unsurprising, given vital signs measure the most critical functions of the human body^38^. Fortunately, vital signs are regularly recorded, either through automatic measurements or regular manual measurements. These advantages mean that vital signs and statistics derived from them are ideal features for use in mortality prediction.

Another important factor in the real-world success of a NN for medical prediction problems is the adoption of clinicians. In recent years, many studies have identified the need for artificial intelligence to be interpretable, especially for healthcare applications^39–42^. Primarily, interpretability involves making the system easier to understand and therefore to trust. There are many ways to achieve this, including selecting features that are simple to understand from the perspective of domain experts^41^.

As such, this paper aims to develop a neural network approach for mortality using straightforward features with clear ties to patient health. Our work contributes to the literature through the development of the novel Artificial Intelligence Mortality Score (AIMS) scheme, a mortality risk classifier based on a hybridized CNN-LSTM network that uses only age, gender, and statistical parameters derived from a 24-h window of vital sign measurements as features. AIMS is capable of continuously-updating prediction of the risk of mortality within 3-day, 7-day, and 14-day windows. Much of the previous literature focuses on predicting mortality events within the entire stay^15–18^, however the average length of stay in ICU in America is only 3.8 days^4^. Our analysis of the patients from the MIMIC-III database who met our selection criteria revealed that 65% of patients stayed in ICU for $\le$ 3 days, 87% for $\le$ 7 days, and 95% for $\le$ 14 days. The models in the literature that focus on the entire stay would likely form a bias towards data obtained from shorter stays, given that these form the majority of cases. This in turn introduces the risk that models would not perform as well on longer-term patients. However, mortality risk assessment needs to be reliable for even the longest staying patients to ensure that they receive the appropriate care should they begin to stabilize or deteriorate. As our selection of 3-day, 7-day and 14-day windows encompasses the entire stay for the majority of patients, it enables fair comparison to the literature. In clinical practice, it offers the clear advantage of predicting risk within a clear time frame, with the score able to be easily and continuously recalculated continuously throughout the stay so that mortality risk is able to be quantified for all patients at all times, including for those with longer stays in ICU.

The remainder of this paper is structured as follows; Section II describes the methodology used for extracting and processing data from the MIMIC III database, as well as the structure of the AIMS network. Section III presents results and discussion, including comparison to currently used schemes and other novel schemes in the literature. Finally, Section IV concludes the paper and presents recommendations for future work.

## Methodology

### Selection of data

The large amount of data used in this work was obtained from the Medical Information Mart for Intensive Care (MIMIC III) clinical database^43^. The MIMIC III database is comprised of deidentified data from over 60,000 ICU stays, including both adult and neonatal patients.

This study focuses on adult patients admitted to ICU for any reason, and thus the only criterion when selecting patient records was that the patient must be $\ge$ 18 years old. To be able to extract data for 3-day, 7-day, and 14-day mortality risk prediction, we obtained a 14-day window for each patient. Our method for selecting data is outlined as follows:

- Where the patient survived their ICU stay, and their stay exceeded 14 days, the first 14 days of data after ICU admission were obtained;
- Where the patient died during their ICU stay, and their stay exceeded 14 days in length, the 14 days of data prior to their death time were obtained;
- Where the patient stay was shorter than 14 days, all data from ICU admission to discharge from ICU were obtained.

For all patients, some fundamental information was recorded during the data selection process—namely their age, gender, and time of death where applicable.

The events that were obtained from the database for our AIMS scheme were the heart rate (HR), systolic BP (SBP), diastolic BP (DBP), mean arterial pressure (MAP), respiratory rate (RR), blood oxygen levels (${\hbox {SpO}}_{2}$), and temperature. All events matching this description were obtained, as our AIMS scheme depends upon statistical analysis of the variation of events such as HR and temperature. Vital signs were chosen as features for two main reasons: interpretability, and ease of measurement in the ICU. Vital signs are the most fundamental indicator of health, and are readily understood by all healthcare professionals. Most vital signs are easily measured using non-invasive equipment, enabling continuous measurement and thus data streams rich with information. Perhaps the most challenging to measure are BP and RR, with continuous methods currently either invasive or uncomfortable. However, recent research in measuring these parameters has focused on non-invasive, continuous methods^44^, and as such it is likely that data streams for BP and RR measurement will become increasingly data rich as this technology is adopted into clinical practice. Richer data streams enable better quantification of the variability of vital signs, and thus would further improve the predictive performance of our network.

### Feature selection

For the development of our AIMS scheme, features were selected or derived from the commonly recorded parameters in the ICU. The first two features selected are those that provide basic information about the patient: their age and gender. Age is recorded in years as an integer, while gender is recorded as a binary value where ‘1’ and ‘0’ represent female and male patients, respectively.

All other features selected were chosen to represent the vital signs of interest—HR, SBP, DBP, MAP, RR, ${\hbox {SpO}}_{2}$ and temperature. These parameters were regularly recorded in the MIMIC III database, however the recording was often inconsistent with the frequency of measurement varying throughout the patient’s stay. This resulted in highly variant quantities of data available for different patients. However, it has previously been shown that trends in vital signs can assist in identification of clinical deterioration in hospital settings^45^. As such, we apply statistical analysis to quantify the variability of each vital sign over the 24-h window. This has the benefit of representing the inconsistent data within the database in a consistent manner, and also has the secondary benefit of improved computational efficiency.

We limit the acquisition window to 24 h to ensure that the network is considering the patient’s current health status. In our own experimentation, narrower windows reduced performance, while broader windows of 48 h did not significantly improve performance. Additionally, if a wider window were used then the network may be prone to under- or over-estimating the severity of the patient’s current condition based on their previous condition. For example, if all data from admission onward were used, then the patient might remain relatively stable for the first 9 days of their stay before showing signs of deterioration on the 10th day. If a risk window from admission onward was used, then overall the variation in the patients health would appear low, and thus the deterioration may not be noticed. Similarly, a patient who is highly unstable at the start of the admission but stabilises as a result of treatment might incorrectly be identified as a mortality risk. Considering a 24-h risk window avoids this problem, as AIMS could be regularly and automatically recalculated throughout the entire stay, and thus would be more capable of identifying deterioration or stabilisation during long stays.

From the 24-h window, the first and last values were taken to indicate how the vital sign changed from the beginning to the end of the window, while the minimum and maximum values were recorded to show the most extreme events during the window. Mean and median were both chosen to provide an accurate representation of the average event for the vital sign—the use of both helps to reduce the risk of unusual data distribution skewing the result. Finally, the standard deviation (STD) is used to quantify the variability of the events for that vital sign. Where any result was NaN, it was replaced with a ‘numerical NaN’ chosen to be the extremely negative value of − 999. If more than two vital signs were completely absent from a patient’s records, then that record was discarded and not used for training or testing of the AIMS model.

After extracting the relevant data, the final feature array included the fundamental patient information, as well as the variability statistics for each vital sign. This resulted in a total of 51 features, including age, gender, and 7 statistical features for each of the 7 vital signs.

Our model was trained to predict three different cases; risks of mortality within 3 days, 7 days, and 14 days respectively. These models are hereafter referred to as AIMS-3, AIMS-7 and AIMS-14 respectively. These windows were selected to consider both immediate mortality risk, and longer term mortality risk. The feature vectors for risk of mortality within 3-day, 7-day and 14-day, risk windows considered the same features, however they were calculated from varying 24-h windows in each case.

Where the patient survived their ICU stay, the relevant 24-h window used for training and testing the model was always the first 24 h after admission. Where the patient did die, the 24-h window selected was dependent on the length of their stay. Where the patient died within less than the 3-day, 7-day, or 14-day risk period, the first 24 h of data post-admission were used. Where their death occurred after a longer stay than the risk window, then their time of death was set as the end time, and the 24-h prediction window was chosen to start from *(end time—size of the risk window)*. For example, where 3-day mortality risk was considered, the death time would be set as ‘72 h’. Then, 72 h prior to death would be chosen as relative 0, with the prediction window thereafter being data recorded between hours 0 to 24.

After data and feature selection, there were 3 distinct cohorts. This was largely as a result of inconsistencies in data richness, with certain variables not recorded for some patients in different windows. This lead to some patients having data available for one or two risk windows, but not the remainder. The cohorts for AIMS-3, AIMS-7, and AIMS-14 are illustrated in Tables 1, 2 and 3. Ages have been clustered by ranges, as ages exceeding 89 in the MIMIC-III database were set to values exceeding 300 for de-identification purposes^43^.

**Table 1:** Characteristics of patient cohort for AIMS-3.

Characteristic	All patients (n = 51,279)	Survived (n = 45,863)	Died (n = 5416)
Female	22,415 (43.71%)	19,888 (43.36%)	2527 (46.66%)
**Age (years)**			
18–39	4896 (9.55%)	4687 (10.22%)	209 (3.86%)
40–59	14,204 (27.70%)	13,147 (28.67%)	1057 (19.52%)
60–79	21,230 (41.40%)	19,040 (41.51%)	2190 (40.44%)
$\ge$ 80	10,949 (21.35%)	8989 (19.60%)	1960 (36.19%)

**Table 2:** Characteristics of patient cohort for AIMS-7.

Characteristic	All patients (n = 51,455)	Survived (n = 45,863)	Died (n = 5592)
Female	22,483 (43.69%)	19,888 (43.36%)	2595 (46.41%)
**Age (years)**			
18–39	4906 (9.53%)	4687 (10.22%)	219 (3.92%)
40–59	14,244 (27.68%)	13,147 (28.67%)	1097 (19.62%)
60–79	21,305 (41.41%)	19,040 (41.51%)	2265 (40.50%)
$\ge$ 80	11,000 (21.38%)	8989 (19.60%)	2011 (35.96%)

**Table 3:** Characteristics of patient cohort for AIMS-14.

Characteristic	All patients (n = 51,639)	Survived (n = 45,863)	Died (n = 5776)
Female	22,560 (43.69%)	19,888 (43.36%)	2672 (46.41%)
**Age (years)**			
18–39	4916 (9.52%)	4687 (10.22%)	229 (3.96%)
40–59	14,282 (27.66%)	13,147 (28.67%)	1135 (19.65%)
60–79	21,397 (41.44%)	19,040 (41.51%)	2357 (40.81%)
$\ge$ 80	11,044 (21.39%)	8989 (19.60%)	2055 (35.58%)

### Neural network structure

**Figure 1:**
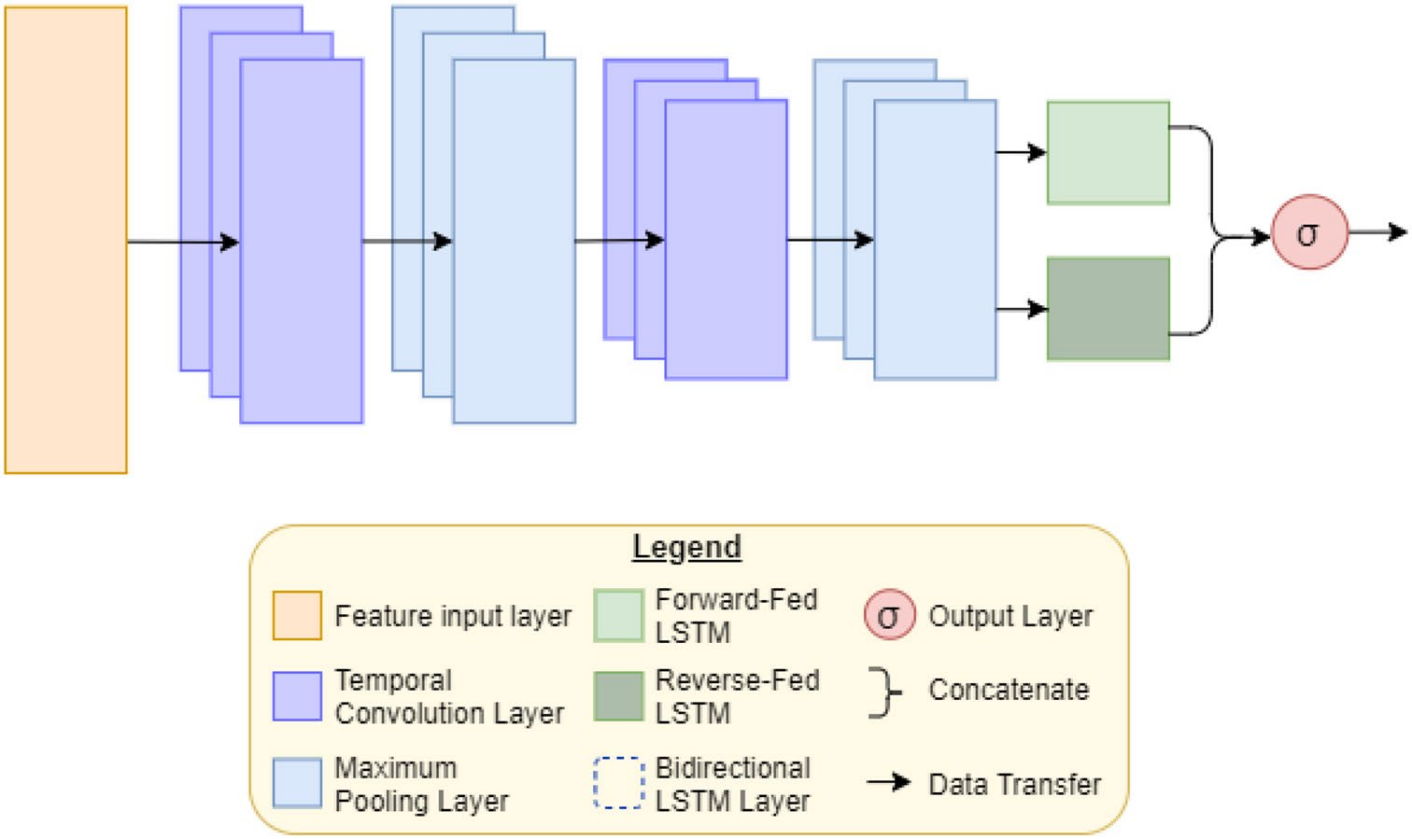
AIMS network structure.

Hybrid NNs offer the advantages of multiple standard NN types. In this application, we develop a hybridized CNN-LSTM network, as shown in Fig. 1. CNNs are widely used to identify patterns and important features, while LSTM networks are known for their “memory”, which enables them to remember which information in a sequence is the most important. Combining the two network structures results in a powerful hybrid NN with strong pattern and sequence recognition abilities, which is highly beneficial in an application where patterns and feature importances are not easily identified.

As shown in Fig. 1, our network includes two temporal (one-dimensional) CNN layers with 128 hidden units each. Both CNN layers utilize rectified linear unit (ReLU) activation, described mathematically as

1 $$\begin{aligned} relu(z) = max(0, z) \end{aligned}$$

The CNN layers can thereafter be mathematically described as follows:

2 $$\begin{aligned} y_{\mathrm{j}}^{\mathrm{i}} = relu\left( \sum _{n=1}^{N}w_{\mathrm{jn}}^{\mathrm{i}}*x_{\mathrm{m}}^{\mathrm{(i-1)}} + b_{\mathrm{j}}^{\mathrm{i}}\right) \end{aligned}$$

where $y_j^i$ represents the output *j*th feature map of the *i*th layer. Convolution is indicated by the $*$ symbol. Weights are denoted using the term $w_{\mathrm{jn}}^{\mathrm{i}}$, which describes the *n*th weight of the *j*th feature map from the $(i-1)$th layer, where $n = 1, \ldots , N$. The parameter $x_{\mathrm{m}}^{\mathrm{(i-1)}}$ represents the outputs of the $(i-1)$th layer, and finally $b_j$ is the *j*th bias term of the *i*th layer. Weight and bias terms are all initialized to zero and updated using the Adam optimization algorithm^46^ throughout training.

Temporal average pooling layers with a pool size of 2 and a stride size of 2 follow each of the CNN layers. This operation sweeps through the output of the CNN layers, taking the average of each pool it sees and outputting that value. Effectively, this downsamples the data by a factor of 2, helping prevent overfitting of the network. Average pooling layers are denoted in Fig. 1 as AvgPool-1 and AvgPool-2, respectively.

A bidirectional LSTM layer with 128 hidden units follows the final temporal average pooling layer. Bidirectional LSTMs (BiLSTMs) have the same mathematical structure as unidirectional LSTMs, but data is passed through the network in both the original and reversed orders. This allows for learning from both past and future values in the sequence. The results of both the forward and reversed passes are then concatenated to form the final output. The mathematical theory of LSTM networks is described by Hochreiter et al.^47^.

The final layer of our AIMS network is a simple densely-connected unit utilizing sigmoid activation, which outputs a value between 0 and 1. If the result is < 0.5, the network predicts that the patient will survive. Conversely, if the result is $\ge$ 0.5, the network predicts that the patient will die. The further away from 0.5 the output is, the more confident the network is in its prediction, and higher confidence typically corresponds with higher accuracy.

For the purposes of training and testing the performance of AIMS, thresholds are used to predict either ‘mortality’ or ‘no mortality’. However, in terms of interpreting this result in a clinical sense, the overall prediction of ‘mortality’ or ‘no mortality’ could be provided alongside a confidence metric that indicates how certain the neural network was of its prediction. The raw 0-1 value outputted by the final layer of the network indicates the level of confidence the network has in its prediction. This confidence metric could be modified for easier understanding by the clinicians using the following equation:

3 $$\begin{aligned} Confidence \; Percentage = \frac{|0.5 - output|}{0.5} \times 100 \end{aligned}$$

Using this equation, a score of 0.14 would be interpreted as ‘no mortality—72% confident’ while a score of 0.78 would be interpreted as ‘mortality—56% confident’. This easy-to-understand strategy would further increase the likelihood that clinicians would trust the model, as they would be able to better understand the severity of the patient’s condition and it would be clearer that AIMS is not simply making binary decisions with full confidence. This metric would also give clinicians more insight into the path of treatment that would be most appropriate. For example, a patient who was predicted as ‘mortality—96% confident’ may require more rapid and extreme treatment than a patient who was predicted as ‘mortality—2% confident’; effectively on the cusp of the two prediction classes.

### Training and testing the algorithms

To train and test the proposed AIMS network, we used stratified *k*-fold cross-validation with 10 folds. Using this method, the data is split in 10 different ways, with all data being used as the testing set during one fold. Stratification ensures that each class is represented roughly equally in all splits. Cross-validation using *k*-fold gives a more realistic idea of the performance of a network.

After preprocessing, records from 51,279 unique patient stays were available for use in training and testing AIMS-3. There were slightly higher record numbers of 51,455 and 51,639 for AIMS-7 and AIMS-14 respectively. The slight differences in record numbers are caused by a higher degree of missingness in some windows, leading to more data that was excluded in those cases. As we were using 10 folds for cross validation, 10% of the data was used for testing purposes and thus was unseen to the model for that fold. A further 80% of the data was used as the training set for AIMS, while the final 10% was used for validation, which improves fine-tuning of hyperparameters and allows for assessment of the “best” model during training.

The data available for this task was highly unbalanced, with mortality events only occurring in up to 11.19% of cases. To ensure that the model did not achieve high accuracy simply by overfitting to the majority class, a weighting of 9 was placed on the importance of learning the minority case. This value is reflective of the mortality rate within the ICUs included in the MIMIC-III database; it was chosen based on the fact that there were approximately 9 non-mortality cases to each mortality case within each cohort. This weighting ensured that the network considered the two classes to be equally important, and placed approximately equal emphasis on accurately predicting both mortality and survival. If the network is to be trained on an ongoing basis in the future, this weighting may need to be adjusted based on the mortality rates within the training set of data, but this could be done programmatically.

For each fold, the AIMS model was trained over 100 epochs with a batch size of 1024. These values were found to be optimal for ensuring that the model is capable of generalizing well, rather than overfitting to the training data. Binary crossentropy was used as the loss function, due to its clear suitability for this binary classification problem. For each fold, the “best” model weights were determined to be those that resulted in the lowest validation set loss during the 100 epochs of training; these weights were saved and used to test the model. The loss function used was binary cross-entropy, due to the binary classification nature of the model.

## Results and discussions

**Figure 2:**
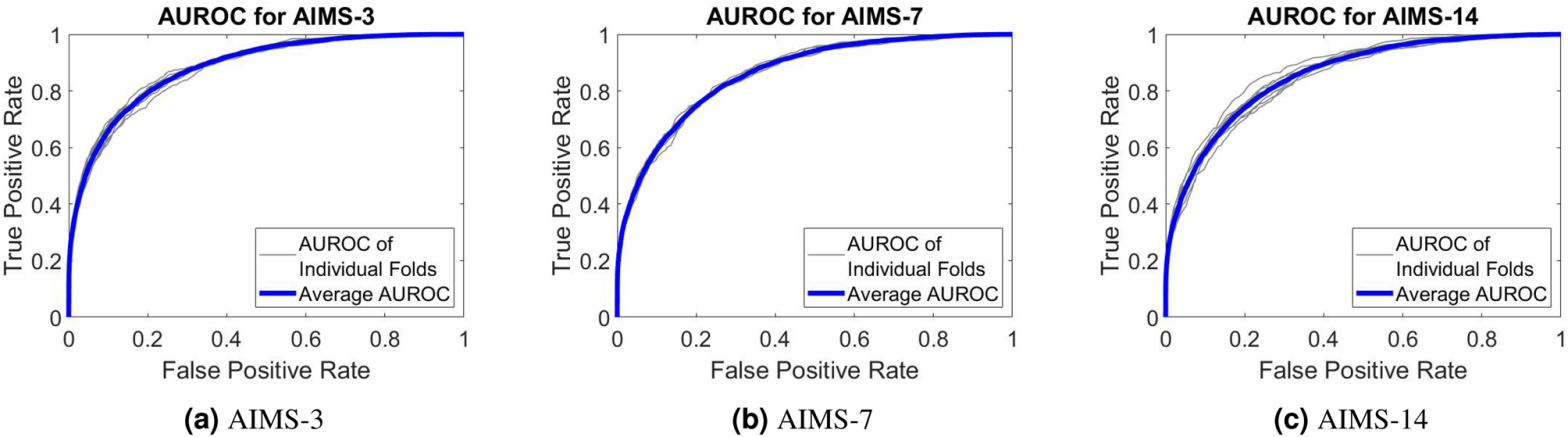
Average ROC and ROC of each fold for 10-fold cross validation.

Following the training and testing of AIMS-3, AIMS-7 and AIMS-14 with 10-fold cross-validation, an extensive statistical analysis was conducted to assess the performance. The most commonly used metric in assessing the performance of a diagnostic tool is AUROC, which plots the true positive rate against the false positive rate. Figure 2a,b illustrate the receiver-operator curves (ROCs) for each of the trained networks. It is clear from these figures that the ROC curve is highly consistent across all 10 folds, with none deviating far from the calculated average. This cross-validation confirms that the results obtained from the AIMS-3, AIMS-7, and AIMS-14 models are a realistic representation of how the network would perform in reality.

**Figure 3:**
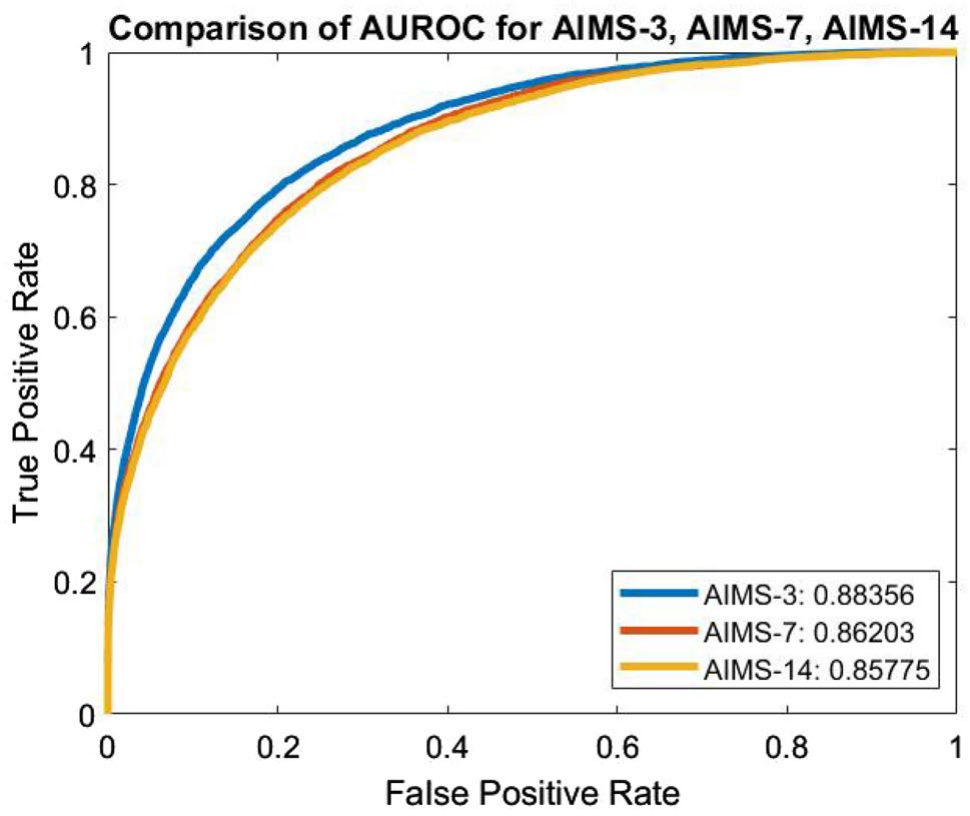
Comparison of ROCs for all AIMS schemes.

Figure 3 further illustrates the differences between the ROC curves for the three networks. As can be observed from this figure, the AIMS-3 model achieves the highest AUROC, followed by AIMS-7 and then AIMS-14. This is a largely unsurprising result as AIMS-3 predicts the risk of death within the shortest window of 3 days. However, this figure also clearly demonstrates that all three models have high AUROC, meaning that they are able to distinguish between mortality and non-mortality cases very well.

A numerical summary of the AUROC results obtained across the 10-fold cross-validation of each model is presented in Table 4. This table highlights the minimum, maximum, and average AUROC obtained across the 10 folds when training each of the three models. These values again indicate the strong consistency across all 10 folds of cross-validation, further suggesting that this model is realistic and suitable for use as a diagnostic tool. The highest variance in AUROCs across folds is seen in AIMS-14, which is to be expected given that accurate prediction becomes more challenging across longer windows. However, the variance is still very low and all folds achieved strong AUROC values.

**Figure 4:**
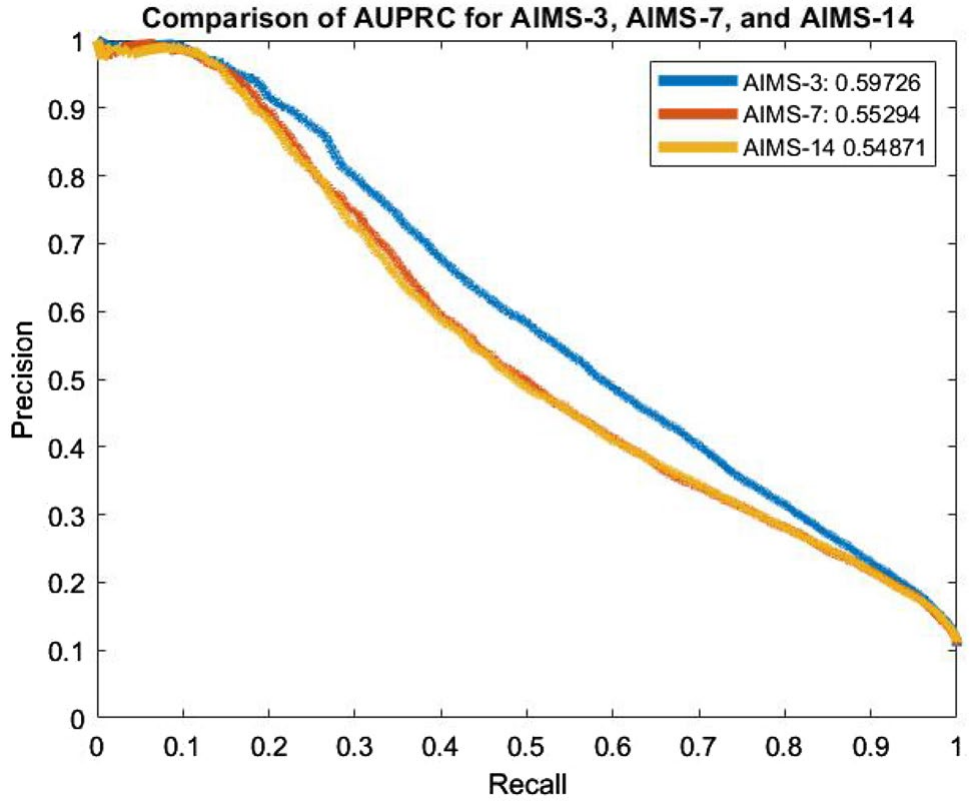
Comparison of PRCs for all AIMS schemes.

**Table 4:** AUROC statistics over 10 folds.

Model	AUROC		
	Minimum	Average	Maximum
AIMS-3	0.8741	0.8835	0.8926
AIMS-7	0.8587	0.8619	0.8676
AIMS-14	0.8399	0.8577	0.8826

An alternative metric to the AUROC considered by some works in previous literature is the area under the precision-recall curve (AUPRC). This metric can be useful where data is imbalanced, as it was within our chosen database. Fig. 4 illustrates the precision-recall curves (PRCs) for each of the AIMS schemes. As is evident in Fig. 4, each of the AIMS models performs strongly and is well above the baseline. Once again, we see that AIMS-3 has the best curve, achieving an average AUPRC of 0.5973 across 10 folds. Meanwhile AIMS-7 and AIMS-14 achieve AUPRCs of 0.5529 and 0.5487, respectively. This again indicates that there is more certainty regarding mortality risk prediction for smaller windows of time.

Other features that are important for any classification problem are accuracy (ACC), specificity or true negative rate (TNR), and sensitivity or true positive rate (TPR). ACC, TNR and TPR provide insight into the overall accuracy, the accuracy for the negative class, and the accuracy for the positive class, respectively. We summarise these parameters, as well as those of AUROC and AUPRC, in Table 5. Each of the presented value is the average, taken across the 10 folds of cross-validation.

**Table 5:** Results obtained by AIMS.

Model	ACC (%)	TNR	TPR	AUROC	AUPRC
AIMS-3	80.07	0.802	0.792	0.884	0.597
AIMS-7	77.07	0.770	0.780	0.862	0.553
AIMS-14	76.22	0.765	0.779	0.858	0.549

Table 5 clearly shows that each of the networks achieves high accuracy. High accuracy alone cannot be used to assess the results where data is so heavily imbalanced, so sensitivity (TPR) and specificity (TNR) are also considered. Both of these parameters are measured between 0 and 1. High sensitivity and specificity indicate the ability of the network to correctly predict the mortality and non-mortality events, respectively. For each of the AIMS models, TPR and TNR are nearly equal, indicating that they can predict both the mortality and non-mortality cases with similar accuracy. Once again, AIMS-3 performs best in all categories, but AIMS-7 and AIMS-14 still show strong performance despite their longer prediction window.

### Comparison to previous works

After performing thorough statistical analysis on AIMS-3, AIMS-7 and AIMS-14, we now consider how these networks perform compared to other state-of-the-art systems presented in the literature. The comprehensive Table 6 compares our three AIMS schemes to schemes presented by several recent papers. Unfortunately, many papers only presented AUROC, but where other metrics were available we have included these for comparison also. Accuracy and TNR have not been included, as these have unfortunately not been presented in any of the works we consider.

**Table 6:** Performance of AIMS-3, AIMS-7, AIMS-14 and other schemes from the literature.

	No. features	Description of features	Measurement window (h)	TPR	AUROC	AUPRC
Alves^15^	37	Vital Signs, Laboratory Results	48 (from admission)	–	0.836	–
Delahanty^18^	17	APR-DRG Codes, MS-DRG Cost Index, GCS, Vital Signs, Laboratory Results	48 (24 h pre- and post-ICU admission)	–	0.94	–
Deliberato^13^ (Best Model)	14	Vital Signs, Demographics, GCS	Varies—1 h from admission, plus pre-admission data and SAPS-II	–	0.84	–
Deliberato^13^ (Vitals Model)	6	Vital Signs	1 (from admission)	–	0.65	–
Johnson^17^	148	Vital Signs, GCS Laboratory Results	24 (from admission)	–	0.927	–
Thorsen-Meyer^32^	44	SAPS-III features (Vital Signs, GCS, Laboratory Values, Comorbidities, Demographics, Patient History)	Various (from admission)	–	0.73–0.88	–
Miao^19^	32	Demographics, Comorbidities, Laboratory Values, Medications	N/A—used first measurements after admission	–	0.821	–
Yu^20^	Varies	Bag-of-words representation	48 (any window)	–	0.8854	0.3184
Yu^14^	15	Vital Signs, GCS, Laboratory Results	24 (from admission)	0.503	–	0.520
Zahid^16^	79	Vital Signs, Laboratory Results, Demographics, GCS	24 (from admission)	–	0.86	–
AIMS-3	51	Age, Gender, Vital Signs	24 (any window)	0.792	0.884	0.597
AIMS-7	51	Age, Gender, Vital Signs	24 (any window)	0.780	0.862	0.553
AIMS-14	51	Age, Gender, Vital Signs	24 (any window)	0.779	0.858	0.549

We include columns presenting the number of features, measurement window, and description of features. The description of features column is used to broadly explain what types of features were used by each network. ‘Vital signs’ refers to raw vital signs and any statistics derived from them, ‘GCS’ is the manually determined Glasgow Coma Score, ‘demographics’ refers to information about the patient’s background, ‘comorbidities’ are existing diagnosed conditions, and ‘medications’ are those administered during the ICU stay. ‘Laboratory results’ refers to results obtained from blood, urine, and other laboratory analysis, including but not limited to: bilirubin, creatinine, hematocrit, blood urea nitrogen, white blood cell count, and many more.

Our AIMS-3 and AIMS-7 networks exceed the performance of all other schemes except those presented by Johnson et al.^17^, Delahanty et al.^18^, Thorsen-Meyer et al.^32^, and K. Yu et al.^20^ in all measured metrics. Compared to the scheme presented by K. Yu et al.^20^, our AIMS-3 scheme achieves an AUROC that is less than 0.002 lower, while AIMS-7 and AIMS-14 achieve slightly lower AUROCs. However, our models perform much more strongly in terms of the AUPRC. This indicates that our models have far stronger precision and recall, which in turn indicates a higher accuracy, and stronger performance on the mortality event class. Additionally, our scheme depends on far simpler features that are easily interpreted by the user.

The model proposed by Thorsen-Meyer et al.^32^ for 90-day mortality from admission time was based on iterative improvement to the measurement throughout the hospital stay. It is based on the same features as SAPS-III, with these features recalculated every hour and used to update the model’s prediction. At admission, the AUROC was at its lowest − 0.73. This then steadily increased throughout the stay, with AUROC reaching 0.82 after 24 h, then 0.85 after 72 h, and finally 0.88 at the time of discharge from ICU. This is an interesting approach for long-term mortality prediction, however it differs from our own scheme in several ways. Firstly, our scheme provides mortality risk prediction within shorter windows, which is more suitable for supporting immediate treatment decisions. Accurate prediction of 90-day mortality is certainly commendable, but the width of the window would limit the usefulness in making treatment decisions during a patient’s stay. Secondly, the dependence of the Thorsen-Meyer et al. model^32^ on SAPS-III parameters introduces a higher burden on healthcare workers, with a number of pathological tests needing to be constantly re-run to keep this information up-to-date. Our scheme uses only vital signs, which are simple to record even without automatic equipment. As such, our scheme would place less additional burden on healthcare providers and would be more suitable for low-resource hospitals. Finally, the Thorsen-Meyer et al. model^32^ has substantially lower AUROC during the early stages of admission, reaching only 0.82 with 24 h of data. All three of our AIMS schemes achieve a higher AUROC using 24 h of data. Furthermore, within 24 h our AIMS-3 scheme achieves a marginally better AUROC than that of the Thorsen-Meyer et al. model^32^ at time of discharge. Overall, the AIMS scheme would be more suitable for short-term mortality prediction in ICU environments.

The AUROC of our strongest network, AIMS-3, also compares favourably to the high-performing scheme presented by Johnson et al.^17^. Unfortunately there are no other metrics presented in this paper to which we can compare. However, it is clear that this model depends on a feature vector nearly three times larger than our own. Additionally, the feature vector used by Johnson et al.^17^ depended heavily upon laboratory values. Of the 148 variables considered, only 20 were vital signs—the rest were the results of laboratory tests and the GCS. As such, it would be extremely difficult to implement this scheme in reality, and even more challenging to update it regularly during the stay, as medical professionals would have to undertake the laborious task of extensive data entry. Meanwhile, our scheme depends primarily on vital signs that are either automatically recorded or manually measured simply and regularly, greatly reducing the demand on healthcare workers.

Minimal statistics were presented by Delahanty et al.^18^, but they do achieve the highest AUROC of the papers we consider. Unfortunately, it has many limitations. While it depends on 17 features directly, three of those features are based on manual diagnosis. As previously discussed, the APR-DRG Risk of Mortality and APR-DRG Severity of Illness are determined based on the diagnosis of the patient, which requires that all diagnoses are known, and also that the hospital uses this particular coding scheme. The scheme also demonstrated strong dependency on MS-DRG codes to determine the Medicare cost-weight index, and these codes are certainly not used in all hospitals. Despite depending on 17 features directly, the dependency of the scheme presented by Delahanty et al.^18^ on these 3 diagnoses-based features means that there is a much greater true dependence based on the many parameters that are used to determine the diagnosis. Additionally, the scheme presented Delahanty et al.^18^ depends on a 48-h window including 24 h pre-ICU admission and 24 h following admission. Therefore, it could not be used in any window other than at ICU admission, and would likely not perform well where the patient is admitted directly to ICU without having first stayed elsewhere in the hospital. Conversely, our AIMS schemes depend on just 24 h of data, and can be easily and automatically updated throughout the patient’s stay.

Overall, our AIMS schemes—particularly AIMS-3—perform strongly as opposed to the comparative schemes in the literature. Two papers—those by Johnosn et al.^17^ and Delahanty et al.^18^ achieved higher AUROC, but presented no other statistics. Additionally, there are strong limiting factors that would prevent their adoption into healthcare environments. Meanwhile, our AIMS networks depend solely upon statistics derived from vital signs and two simple demographics—age and gender. These parameters are regularly recorded in ICUs, often automatically. Additionally, the selection of vital signs as parameters would ensure that even low-resource healthcare environments would be able to utilise our scheme. The ease of measurement would also be extremely valuable in times of crises where a high number of patients may be admitted to intensive care, such as following a natural disaster or during a pandemic like COVID-19. Other schemes in the literature that rely on time-consuming and laborious collection of pathology results and/or patient histories would place high burden on an already strained system, making them challenging and impractical to use in such situations.

Our AIMS models also have the advantage of being easy to calculate in any 24-h window due to the regular and often continuous recording of vital signs. This is a significant advantage over other schemes that have only considered calculation of mortality during a single window immediately following admission. We therefore conclude that our scheme is a strong candidate for predicting real-time mortality in ICU environments, however we acknowledge that this study has been conducted retrospectively on a single popular ICU database. We aim to further verify the performance of AIMS through clinical trials, with the aim of ensuring that it will perform as strongly on other patient populations. Further improvements will be made as necessary to ensure that the AIMS scheme performs equally well across all populations.

## Conclusion

In this paper, we have presented AIMS, a hybrid neural network structure that combines temporal convolution layers with long short-term memory layers. We have then trained and tested three instances of AIMS: AIMS-3, AIMS-7, and AIMS-14, which predict the risk of a mortality event within the following 3, 7, and 14 days, respectively.

AIMS-3 was the highest performing instance of the network, however AIMS-7 and AIMS-14 also perform strongly and compare well to other schemes in the literature. All three schemes could be used simultaneously in hospitals, giving healthcare workers a clear picture of both short-term and longer-term mortality risk to the patient.

The AIMS scheme is dependent only on age, gender, and statistics derived from vital signs. These features are all readily and regularly recorded in ICU environments, with minimal effort required by healthcare workers. The simplicity of the features chosen also ensures that AIMS could be recalculated on a continuous and automatic basis during a patient’s stay. This would provide invaluable information about whether a patient is responding to treatment or not, thus allowing medical professionals to modify their treatment plan more readily. Furthermore, the simplicity of both inputs and outputs to AIMS improves the interpretability of the overall model, thus improving the likelihood that healthcare providers would place their trust in its predictions.

One limiting factor for this work was that the MIMIC-III records for individual patients are not equally data-rich for all windows considered. This leads to some patients being included in the cohort for one or two schemes, but not the remainder. In turn, this prevented robust analysis of stability between the systems—that is, analysis of how similar the predictions of AIMS-3, AIMS-7, and AIMS-14 were for a single patient. We aim to address this in the clinical trial phase, where we will be able to ensure that data is recorded with consistent frequency throughout trials.

Overall, AIMS is an easy-to-interpret and powerful tool for mortality risk prediction in the ICU. The results presented in this paper indicate that the three AIMS networks may be suitable for clinical implementation. In our own future works, we aim to conduct clinical trials using the AIMS networks to further analyse and improve upon its performance. We will also seek to assess the interpretability of the system during the clinical trial process, improving upon it as necessary.
